# Statistical Analysis of Zebrafish Locomotor Behaviour by Generalized Linear Mixed Models

**DOI:** 10.1038/s41598-017-02822-w

**Published:** 2017-06-07

**Authors:** Yiwen Liu, Ping Ma, Paige A. Cassidy, Robert Carmer, Gaonan Zhang, Prahatha Venkatraman, Skye A. Brown, Chi Pui Pang, Wenxuan Zhong, Mingzhi Zhang, Yuk Fai Leung

**Affiliations:** 1 Department of Statistics, University of Georgia, 101 Cedar St, Athens, GA 30602 USA; 2 Department of Biological Sciences, Purdue University, 915 W. State Street, West Lafayette, IN 47907 USA; 3 Department of Statistics, 250 N University Street, Purdue University, West Lafayette, IN 47907 USA; 4 Department of Ophthalmology and Visual Sciences, Chinese University of Hong Kong, Hong Kong, Hong Kong; 5 Joint Shantou International Eye Center, Shantou University & the Chinese University of Hong Kong, Shantou, China; 6 Department of Biochemistry and Molecular Biology, Indiana University School of Medicine Lafayette, 625 Harrison Street, West Lafayette, IN 47907 USA; 7 Purdue Institute for Integrative neuroscience, 610 Purdue Mall, Purdue University, West Lafayette, IN 47907 USA; 8 Purdue Institute for Drug Discovery, 610 Purdue Mall, Purdue University, West Lafayette, IN 47907 USA

## Abstract

Upon a drastic change in environmental illumination, zebrafish larvae display a rapid locomotor response. This response can be simultaneously tracked from larvae arranged in multi-well plates. The resulting data have provided new insights into neuro-behaviour. The features of these data, however, present a challenge to traditional statistical tests. For example, many larvae display little or no movement. Thus, the larval responses have many zero values and are imbalanced. These responses are also measured repeatedly from the same well, which results in correlated observations. These analytical issues were addressed in this study by the generalized linear mixed model (GLMM). This approach deals with binary responses and characterizes the correlation of observations in the same group. It was used to analyze a previously reported dataset. Before applying the GLMM, the activity values were transformed to binary responses (movement vs. no movement) to reduce data imbalance. Moreover, the GLMM estimated the variations among the effects of different well locations, which would eliminate the location effects when two biological groups or conditions were compared. By addressing the data-imbalance and location-correlation issues, the GLMM effectively quantified true biological effects on zebrafish locomotor response.

## Introduction

Zebrafish are widely used in neurobehavioural research because this model confers several unique advantages. For example, zebrafish have high fecundity and routinely lay hundreds of embryos when mated in pairs. These embryos are also small and develop quickly into freely-swimming larvae in three to four days, which makes simultaneous tracking of their locomotor behavior under different experimental conditions straightforward. This approach has indeed generated data that provide new insights into neurobiology^1–15^, pharmacology^3, 5–7, 9–12, 16–18^ and toxicology^19–27^. Nonetheless, the resulting data are high-dimensional and complex, and require new methods of statistical analysis to unveil critical information about the underlying neurobehaviour.

To illustrate the analytical challenges, we will focus on one popular approach for high-throughput behavioural analysis: the visual motor response (VMR). This is an instantaneous locomotor response displayed by zebrafish larvae upon drastic light onset or offset^4, 28–30^. In a typical VMR experiment, zebrafish are arranged in a 96-well plate, isolated from environmental light in a lightproof chamber, and stimulated by controlled white light. Their activities are recorded and summarized as the number of pixels moved in successive frames or as absolute displacement^31^. The resulting VMR data have two major features. First, the distribution of the larval activity would likely deviate from a Gaussian distribution because many larvae display little or no movement. This deviation creates data imbalance, and may pose challenges to statistical analysis since most traditional methods rely on the assumption of a Gaussian distribution. Second, the larval activities are observed repeatedly over time and in groups, such as larvae from the same location of the plate. Different locations in a well plate may have different effects on larval activity. Treating all location equally ignores not only the variations among those location effects, but also the correlations of larvae in the same location of the plate. This variation accounts for the unobserved heterogeneity of the data, while the correlation between larvae in the same wells would result in correlated samples. These repeated observations must be properly handled during data analysis.

These data features pose challenges to analyzing VMR or similar locomotor data by traditional approaches. For example, the t-test and analysis of variance (ANOVA) are often used to compare data between two groups, and three or more groups respectively. These tests have been implemented in analyzing similar locomotor data^32^ despite several limitations: the t-test has a higher Type I error rate when more comparisons are performed, whereas both t-test and ANOVA do not handle the time-dependency issue as commonly observed in time-series data. This time-dependency issue is often tackled by repeated-measures ANOVA^13, 21, 33, 34^, a variant of ANOVA that can handle dynamical changes in behavior and repeatedly measured samples that are correlated in time. This analysis, however, assumes that the variances of the differences between group combinations are equal, an assumption that is hardly satisfied in behavioural data. To address these analytical issues, we recently introduced the Hotelling’s T-squared test and multivariate analysis of variance (MANOVA; a multivariate analog of ANOVA) for analyzing locomotor data^31^. Hotelling’s T-squared test not only reduces the Type I error rate compared to the t-test, but it also takes into account the time dependency between repeated measures; whereas MANOVA considers the time dependency and quantifies the effect sizes of variables that contribute to locomotor behaviour. These two methods, however, still treat samples collected in the same location of the plate as independent measurements and do not consider the correlations between them. They also do not address the data-imbalance issue, where the larval activity does not satisfy normality assumption. Several zebrafish behavioural studies used nonparametric tests such as Kruskal-Wallis test and Wilcoxon signed-rank test when the normality test indicated the data were not normally distributed^35–40^. However, simple non-parametric tests have their disadvantages. For example, they cannot make quantitative statements about the difference between two groups. Moreover, non-parametric tests such as Wilcoxon signed-rank also suffer from loss of information, since they only utilize the ranks of the data. They are also less sensitive to differences between groups and require a larger sample size to achieve the same power as parametric tests.

To address these analytical issues, we present an alternative approach for the analysis of locomotor behaviour: the generalized linear mixed model (GLMM). This approach handles binary response variables and can be used to estimate the probability of the binary response based on multiple predictors^41^. It also assumes that the conditional distribution of the response variable is a Bernoulli distribution rather than a Gaussian distribution. When this approach is used to analyze locomotor data, the activity values are transformed into binary responses and encoded as 0 (no movement) and 1 (otherwise). This transformation renders the data less imbalanced. Moreover, different from displacement, the GLMM characterizes the larval activity by the proportion of larvae moved at each second, which represents how active the whole group is. Furthermore, it also treats group-level terms, such as location, as random effects. By controlling for these unobserved covariates, the approach can efficiently estimate the coefficients of other variables. It also adjusted for the lack of independence among the multiple observations for each location. The GLMM was used in this study to analyze a standard VMR dataset that was used previously to develop the Hotelling’s T-squared test^31^. Our results indicate that the GLMM efficiently handled the complex structure of high-throughput behavioral data. This GLMM approach complements the Hotelling’s T-squared test for analyzing VMR data, and these approaches together establish a framework that can be used to analyze behavioural data with a similar structure.

## Materials and Methods

### Experimental data

All experimental data were previously collected, reported^31^ and are accessible from the Harvard Dataverse (http://dx.doi.org/10.7910/DVN/HTXXKW). A summary of the data will be provided here. These data were collected from VMR experiments on three wild-type (WT) zebrafish strains: AB, TL and TLAB. Their VMR were analyzed daily from 3 days post-fertilization (dpf) to 9 dpf, using a standard VMR experimental scheme^4, 14, 15, 18, 28, 31^. In this scheme, the larvae were arrayed in a 96-well plate and dark adapted for 3.5 hrs. They were then subjected to three consecutive trials of light onset (Light-On) and light offset (Light-Off). Each trial session lasted for 30 mins. We also controlled other experimental variables that might affect the results^31^. For example, only healthy larvae were included in the final analysis, and the same type of 96-well plate was used for all experiments. We also ran all strains separately. The reasons of this experimental design are as follows. Firstly, to quantify the variations among wells (locations), the biological groups (i.e. strains) should not be confounded with the technical groups (i.e. locations). In other words, there was no interaction between strain and location under such circumstance. We could then assume larvae at the same location would have the same location effect, regardless of their biological groups. Secondly, we also conducted biological replicates of each experiment. The estimated strain effect is the “mean effect” of all replicates, which alleviated the variation due to running all strains separately and ensured a valid biological interpretation of the results.

### Statistical Analysis

#### Activity summarization

The VMR dataset used in this study summarized the larval activity as Burst Duration, the fraction of frames in each second that a larva moved^31^. Each frame was compared with the previous one. A larva would be declared moving in a frame if it moved more than a preset threshold. However, these summarized Burst-Duration values were imbalanced since a large number of zebrafish larvae displayed little or no movement at all. This data-imbalance issue was handled by transforming the Burst-Duration values into binary responses: all non-zero values were transformed to 1 or 0 otherwise.

#### Data modeling and statistical inference

In the VMR experiment, zebrafish larvae would display very drastic movement after sudden light change. We previously used their activity data from the 30-second period after each light change to develop analytical tools for VMR data^31^. In this study, we used the same period of time for analysis. All explanatory variables in our models are introduced in section Explanatory variables. The effects of these variables are analyzed by GLMM and introduced in section GLMM. All statistical analyses were performed using R software version 3.2.3 (https://www.r-project.org). The analysis scripts are available in the Supplementary file.

#### Explanatory variables

The explanatory variables used in our models include: (1) Strain: AB, TL and TLAB; (2) Stage: 3–9 dpf; (3) Light stimulus: light-onset sessions (Light-On) and light-offset sessions (Light-Off); (4) Time: 1–30 seconds after the light change; (5) Location: The well position in a 96-well plate; and (6) Interactions between the variables (1–5).

#### GLMM

Assume that *y*_*ij*_ is the observation of the *j* th zebrafish larva in group *i* for *j* = 1, …, *n*_*i*_, with *y*_*ij*_ = 1 representing an active zebrafish larva and *y*_*ij*_ = 0 otherwise, and ***x***_***ij***_ as a column vector of values of explanatory variables for this larva. Then, the GLMM^41^ has the following form:

1 $$logit[\,P(\,{y}_{ij}=1|{\gamma }_{i}\,)]={{\boldsymbol{x}}}_{ij}^{T}{\boldsymbol{\beta }}+{\gamma }_{i},$$

where $logit[\,P({y}_{ij}=1|{\gamma }_{i})\,]=\,\mathrm{log}\,[\frac{P({y}_{ij}=1|{\gamma }_{i})}{1-P\,({y}_{ij}=1|{\gamma }_{i}\,)}]$, and ***β*** represents the fixed-effect model parameters. *γ*_*i*_ is the random effect of group *i*, and {*γ*_*i*_} are independent *N*(0, *σ* ^2^). In our studies, the larval location in the 96-well plate was modeled as the random effect. Zebrafish larvae at different locations (i.e. wells) were independent from each other, whereas zebrafish larvae at the same location had the same effect size. Other explanatory variables included the strain and stage of the zebrafish, time, and light stimulus.

## Results

In this study, we used the GLMM to resolve the aforementioned data-analysis issues that were not handled by both traditional analyses and Hotelling’s T-squared test^31^. We focused on the 1^st^ Light-On session (i.e. 1^st^ technical repeat) in this study whenever possible. This selection simplified the analysis, as the 1^st^ Light-On session (i.e. 1^st^ technical repeat) was qualitatively different from the 2^nd^ and 3^rd^ (p < 0.05) due to a difference in the length of the prior dark adaptation^31^. By using one technical repeat, we could effectively compare the analyses of the Hotelling’s T-squared test and GLMM, and illustrate the potential of the GLMM in VMR data analysis. Three examples will be shown below.

### Example 1: Difference in activities of different WT strains during the same time interval

To determine this difference, a model (1) was built on the VMR data of larvae at 6 dpf from 1 to 30 s with the following variables: strain (categorical; *S*), time and its squared term (continuous; *t* and *t* ^2^), their interactions, and random effect of location (*γ*_*i*_). The time variable was centered to have a mean of zero to reduce the degree of multicollinearity. The squared term of time was included since the log odds of moving larvae proportion were not linear across time. For each strain *m* (AB, TL, TLAB), we denoted ${\beta }_{s}^{(m)},{\beta }_{{I}_{1}}^{(m)}$, and ${\beta }_{{I}_{2}}^{(m)}$ as the coefficients of strain, and its interactions with *t* and *t* ^2^ respectively. Each level of the categorical variables was compared with a reference level. For example, AB was the reference level for the variable strain, and its corresponding coefficient was set to zero (i.e. *β*_*s*_ satisfied the constraint ${\beta }_{s}^{(AB)}=0)$. The results of the model were interpreted as log odds of activeness (LOA), defined as

2 $$LOA=\,\mathrm{log}\,\frac{P({y}_{ij}=1|{\gamma }_{i})}{1-P({y}_{ij}=1|{\gamma }_{i})}.$$

The LOA describes the likelihood that the larva in a particular location would move. Its value might be different in different strains. The LOA difference between different strains (dLOA) was deduced by the following formula, using the comparison between TL and AB strains in the same location as an example:

$$LOA(TL)-LOA(AB)={\beta }_{s}^{(TL)}-{\beta }_{s}^{(AB)}+({\beta }_{{I}_{1}}^{(TL)}-{\beta }_{{I}_{1}}^{(AB)})t+({\beta }_{{I}_{2}}^{(TL)}-{\beta }_{{I}_{2}}^{(AB)}){t}^{2}.$$

Since the other basic effects were the same between the two strains at time *t*, they canceled each other. The random effects (*γ*_*i*_) also canceled each other for the same location. The first part of the formula, ${\beta }_{s}^{(TL)}-{\beta }_{s}^{(AB)}$, describes the average shift in LOA between TL and AB independent of time; whereas the second and third parts indicate how dLOA changed over time.

**Figure 1:**
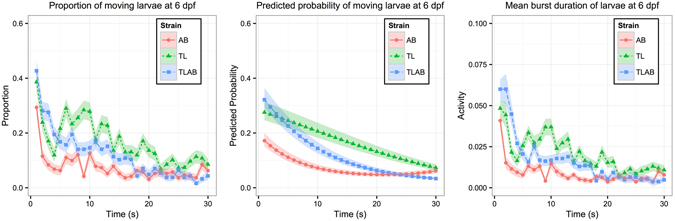
Plots of VMR during the first 30 seconds of Light-On stimulus for WT larvae at 6 dpf. Left panel: Proportions of moving larvae summarized from the data. Y-axis is the proportion of moving larvae and x-axis is time (1–30s). For each strain, the proportions are shown in different colours. The corresponding ribbon represents 1 standard error from the proportion. Middle panel: Predicted probability of moving larvae. Y-axis is the predicted probability of detecting a moving zebrafish larva $\hat{P}({y}_{ij}=1)$ and x-axis is time (1–30s). The predicted probability is shown in a different colour for each strain. The corresponding ribbons represent the lower and upper quartiles. Note that the Y-axes of left and middle panels are the proportion and predicted probability of moving larvae respectively, which can be derived from the LOA, defined as $\mathrm{log}\,[\frac{P({y}_{ij}=1|{\gamma }_{i})}{1-P\,({y}_{ij}=1|{\gamma }_{i}\,)}]$. Right panel: Mean Burst Duration of zebrafish larvae during the same time interval. For each strain, its corresponding ribbon represents 1 standard error from the mean activity. These data were used for the Hotelling’s T-squared tests that are reproduced in Table 2. The sample size in this example is 16560 (AB: 5730; TL: 5280; TLAB: 5550).

The fitting results are shown in Table 1, and the corresponding data are plotted in the left panel of Fig. 1. The effect of strain on LOA was decomposed into two parts. The first part was in the main effect. The LOAs of TL and TLAB strains increased by 1.2551 (p < 0.0001) and 0.5601 (p < 0.0001) respectively when compared with that of AB. Thus, more TL and TLAB larvae tended to move when exposed to a Light-On stimulus. In terms of instant behaviour $({\rm{i}}.{\rm{e}}.\,{\rm{at}}\,t=1)$, there was no significant difference between TL and TLAB larvae. Their LOAs increased by 0.6076 and 0.8342 respectively when compared with that of AB. This can be due to the SNPs in the TL line were associated with dominant genes for higher locomotor activities. The hybrid TLAB line would therefore still display the dominant active phenotype. The second part of strain effect on LOA was in its interaction with time. The dLOA between TL and TLAB had a significant linear pattern over time (2.1698, p < 0.0001); whereas the dLOA between TLAB and AB strains had significant linear (−3.0496, p < 0.0001) and quadratic patterns (−7.9245, p = 0.0330) over time. This trend was also shown by the predicted probability of WT strains that moved during the same time interval (Fig. 1, middle panel). When TL and TLAB strains were exposed to a Light-On stimulus, more of them tended to move compared to AB. TLAB larvae, however, returned to the baseline activity faster than AB and TL larvae, as the proportion of moving TLAB larvae decreased faster than that of AB and TL.

**Table 1:** The GLMM results of Light-On VMR from 1 to 30s for different WT strains at 6 dpf.

Mean (standard error) (p-value*)						
Basic Effects	Intercept *β*_0_		*β*_*t*_		${\beta }_{{t}^{2}}$	
	**−2.9206 (<0.0001)**		**−2.4019 (<0.0001)**		**12.5726 (<0.0001)**	
Strain Effect	Main effect		Interactions with time			
	${\beta }_{s}^{(TL)}-{\beta }_{s}^{(AB)}$	**1.2551 (<0.0001)**	${\beta }_{{I}_{1}}^{(TL)}-{\beta }_{{I}_{1}}^{(AB)}$	−0.8793 (0.05)	${\beta }_{{I}_{2}}^{(TL)}-{\beta }_{{I}_{2}}^{(AB)}$	**−14.7263 (<0.0001)**
	${\beta }_{s}^{(TLAB)}-{\beta }_{s}^{(AB)}$	**0.5601 (<0.0001)**	${\beta }_{{I}_{1}}^{(TLAB)}-{\beta }_{{I}_{1}}^{(AB)}$	**−3.0496 (<0.0001)**	${\beta }_{{I}_{2}}^{(TLAB)}-{\beta }_{{I}_{2}}^{(AB)}$	**−7.9245 (0.0330)**
	${\beta }_{s}^{(TL)}-{\beta }_{s}^{(TLAB)}$	**0.6951 (<0.0001)**	${\beta }_{{I}_{1}}^{(TL)}-{\beta }_{{I}_{1}}^{(TLAB)}$	**2.1698 (<0.0001)**	${\beta }_{{I}_{3}}^{(TL)}-{\beta }_{{I}_{3}}^{(TLAB)}$	−6.8037 (0.05)

^*^p-values of these tests were adjusted using the Benjamini–Hochberg procedure to control for Type I error.

This comparison unveiled new information compared to that obtained from the Hotelling’s T-squared test performed on the same data (Table 2; also plotted accordingly in the right panel of Fig. 1)^31^. In our previous work, the Hotelling’s T-squared test showed that the average activity of every strain across time was different from the others (Table 2; p-values for all pairwise comparison were <0.0001). As a complementary analysis, the GLMM further improves on the explanation of this difference: (1) TL had a larger strain effect (Table 1, main effects); (2) the LOA of TLAB strain decreased faster than that of TL strain during the 30s period; and (3) the LOAs of all strains decreased in significant nonlinear patterns across time (Table 1, interactions with time).

**Table 2:** The Hotelling’s T-squared test of Light-On VMR data used in Table 1.

	AB VS. TL	TL VS. TLAB	AB VS. TLAB
Test statistic* (p-value)	**5.1843 (<0.0001)**	**2.3645 (<0.0001)**	**3.7021 (<0.0001)**

^*^These results are reproduced from ^31^ for comparison.

## Difference in activity of the same strain

### Example 2: Difference in activity of the same strain during light onset and offset

The larvae displayed substantially different activities by the Light-On and Light-Off stimuli. This difference was quantitatively evaluated by GLMM, using VMR data of AB at 6 dpf from 1 to 30s (i.e. after light change) with the following variables: light stimulus (categorical; *L*), time and its squared term (continuous; *t* and *t* ^2^), their interactions, and random effect of location (*γ*_*i*_). For light stimulus *l* (ON or OFF), we denoted ${\beta }_{L}^{(l)},\,{\beta }_{{I}_{1}}^{(l)}$ and ${\beta }_{{I}_{2}}^{(l)}$ as the coefficients of light stimulus and its interactions with *t* and *t* ^2^ respectively.

**Figure 2:**
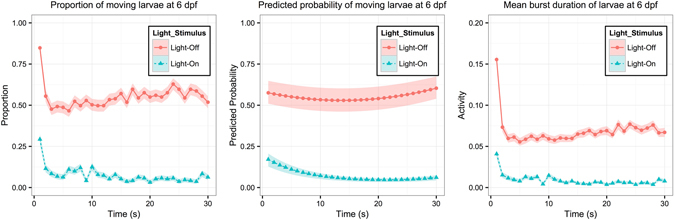
Plots of VMR during the first 30 seconds of the light-stimulus change for AB larvae at 6 dpf. Left panel: Proportions of moving larvae summarized from the data. Y-axis is the proportion of moving larvae and x-axis is time (1–30s). For light onset and offset, the proportions are shown in different colours. The corresponding ribbon represents 1 standard error from the proportion. Middle panel: Predicted probability of moving larvae. Y-axis is the predicted probability of detecting a moving zebrafish larva and x-axis is time (1–30s). The predicted probability is shown in a different colour for each strain. The corresponding ribbons represent the lower and upper quartiles. Note that the Y-axes of left and middle panels are the proportion and predicted probability of moving larvae respectively, which can be derived from the LOA defined as $\mathrm{log}\,[\frac{P({y}_{ij}=1|{\gamma }_{i})}{1-P\,({y}_{ij}=1|{\gamma }_{i}\,)}]$. Right panel: Mean Burst Duration of zebrafish larvae during the same time interval. For light onset or offset, its corresponding ribbon represents 1 standard error from the mean activity. These data were used for the Hotelling’s T-squared tests showed in Example 2. The sample size in this example is 11460 (Light-On: 5730; Light-Off: 5730).

The fitting results are summarized in Table 3, and the corresponding data are plotted in the left panel of Fig. 2. In main effect, the LOA of larvae upon Light-On stimulus was significantly smaller than that upon Light-Off stimulus (−3.0963; p < 0.0001). In other words, fewer zebrafish larvae moved upon Light-On stimulus. The larvae also displayed different decreasing LOA patterns during light onset and offset, as evidenced by the significant dLOAs in the interaction between light stimulus and time (linear term: −2.6759, p < 0.0001; quadratic term: 8.4131, p < 0.0043).

**Table 3:** The GLMM results of VMR data from 1 to 30s for AB larvae at 6 dpf.

Mean (p-value)						
Basic Effects	Intercept *β*_0_		*β*_*t*_		${\beta }_{{t}^{2}}$	
	**0.1268 (0.0470)**		0.2496 (0.1888)		**4.3020 (0.0028)**	
Light Stimulus Effects	Main Effect		Interaction with Time			
	${\beta }_{L}^{(ON)}-{\beta }_{L}^{(OFF)}$	**−3.0963 (<0.0001)**	${\beta }_{{I}_{1}}^{(ON)}-{\beta }_{{I}_{1}}^{(OFF)}$	**−2.6759 (<0.0001)**	${\beta }_{{I}_{2}}^{(ON)}-{\beta }_{{I}_{2}}^{(OFF)}$	**8.4131 (0.0043)**

The GLMM results were more informative compared to those obtained from the Hotelling’s T-squared test on the same data. Previously we showed that the larval activity differed upon light onset and offset by Hotelling’s T-squared test (p < 0.0001). We further explained this difference using the results of the GLMM (Fig. 2, middle panel; Table 3, main effects), which showed that the proportion of active larvae was larger during light onset than offset. The GLMM also revealed that larval activity decreased at a different rate upon light onset and offset (Table 3, interaction with time). The results from the two analyses therefore complemented each other and described different aspects of the larval activity.

### Example 3: Difference in activities of larvae at different developmental stages

The VMR data were collected from 3 to 9 dpf, when the larvae were developing. Their maturation would alter the locomotor behaviour^14, 31^. This developmental difference was modeled by the GLMM, using the VMR data of AB larvae. The model focused on the 1^st^ technical repeat of the Light-On stimulus from 1 to 30 s. It comprised the following variables: stage (categorical; *G*), time and its squared term (continuous; *t* and *t* ^2^), their interaction s, and random effect of location (*γ*_*i*_). For stage *k* (3, …, 9), we denoted ${\beta }_{G}^{(k)},\,{\beta }_{{I}_{1}}^{(k)}$ and ${\beta }_{{I}_{2}}^{(k)}$ as coefficients of stage and its interactions with *t* and *t* ^2^ respectively.

**Figure 3:**
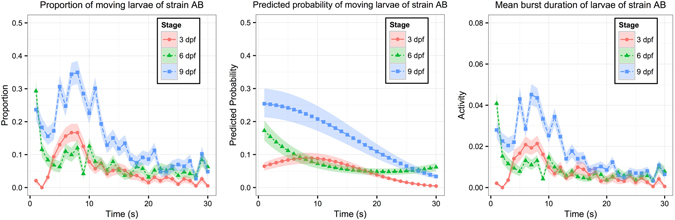
Plots of VMR during the first 30 seconds of Light-On stimulus for AB larvae at 3, 6 and 9 dpf. Left panel: Proportions of moving larvae summarized from the data. Y-axis is the proportion of moving larvae and x-axis is time (1–30s). For different stages, the proportions are shown in different colours. The corresponding ribbon represents 1 standard error from the proportion. Middle panel: Predicted probability of moving larvae. Y-axis is the predicted probability of detecting a moving zebrafish larva and x-axis is time (1–30s). The predicted probability is shown in a different colour for each strain. The corresponding ribbons are the lower and upper quartiles. Note that the Y-axes of left and middle panels are the proportion and predicted probability of moving larvae respectively, which can be derived from the LOA defined as $\mathrm{log}\,[\frac{P({y}_{ij}=1|{\gamma }_{i})}{1-P\,({y}_{ij}=1|{\gamma }_{i}\,)}]$. Right panel: Mean Burst Duration of zebrafish larvae during the same time interval. For each stage, its corresponding ribbon represents 1 standard error from the mean activity. These data were used for the Hotelling’s T-squared tests that are reproduced in Table 5. The sample size in this example is 39900 (3 dpf: 5760; 4 dpf: 5760; 5 dpf: 5760; 6 dpf: 5730; 7 dpf: 5670; 8 dpf: 5640; 9 dpf: 5580).

The fitting results of 3, 6 and 9 dpf are summarized in Table 4, and the corresponding data are plotted in the left panel of Fig. 3. The effect of stage on LOA was decomposed into two parts. The first part was the main effect. The LOA of AB larvae was significantly larger at 9 dpf than 3 and 6 dpf (0.9693, p < 0.0001; 1.1838, p < 0.0001). Thus, fewer zebrafish larvae tended to move upon light onset at 3 and 6 dpf than at 9 dpf (Fig. 3, middle panel). The second part of stage effect on LOA was in the interactions of stage effect with time. The LOAs of larvae at all stages decreased in nonlinear patterns, and were quite different from each other (Table 4, interactions with time). At 3 dpf, the LOA gradually increased upon light onset, and then gradually decreased (Table 4, *β*_*t*_ = −5.6808; ${\beta }_{{t}^{2}}$ = −23.7371). At 6 dpf, however, the LOA drastically increased upon light onset and then gradually decreased (Table 4, ${\beta }_{t}+{\beta }_{{I}_{1}}^{(6)}$= −2.4029; ${\beta }_{{t}^{2}}+{\beta }_{{I}_{2}}^{(6)}$= 12.5912). At 9 dpf, the LOA decreased in both linear and nonlinear pattern over time, and its nonlinear term was significantly different than that at 3 dpf (Table 4, ${\beta }_{{I}_{2}}^{(9)}-{\beta }_{{I}_{2}}^{(3)}$=14.7728, p < 0.0001).

**Table 4:** The GLMM results of Light-On VMR from 1 to 30s for AB larvae at different stages.

Mean (p-value*)					
Intercept *β*_0_		*β*_*t*_		${\beta }_{{t}^{2}}$	
**−2.7059 (<0.0001)**		**−5.6808 (<0.0001)**		**−23.7371 (<0.0001)**	
Main effect		Interactions with time			
${\beta }_{G}^{(6)}-{\beta }_{G}^{(3)}$	−0.2142 (0.0528)	${\beta }_{{I}_{1}}^{(6)}-{\beta }_{{I}_{1}}^{(3)}$	**3.2779 (<0.0001)**	${\beta }_{{I}_{2}}^{(6)}-{\beta }_{{I}_{2}}^{(3)}$	**36.3283 (<0.0001)**
${\beta }_{G}^{(9)}-{\beta }_{G}^{(3)}$	**0.9693 (<0.0001)**	${\beta }_{{I}_{1}}^{(9)}-{\beta }_{{I}_{1}}^{(3)}$	0.9164 (0.1445)	${\beta }_{{I}_{2}}^{(9)}-{\beta }_{{I}_{2}}^{(3)}$	**14.7728 (<0.0001)**
${\beta }_{G}^{(9)}-{\beta }_{G}^{(6)}$	**1.1838 (<0.0001)**	${\beta }_{{I}_{1}}^{(9)}-{\beta }_{{I}_{1}}^{(6)}$	**−2.3585 (<0.0001)**	${\beta }_{{I}_{2}}^{(9)}-{\beta }_{{I}_{2}}^{(6)}$	**−21.5533 (0.0034)**

^*^p-values of these tests were adjusted using the Benjamini–Hochberg procedure to control for type I error.

These results again unveiled new information and complemented the findings from the Hotelling’s T-squared test that were thoroughly discussed in our previous study^31^. For example, in our previous work, the Hotelling’s T-squared test showed a significant difference in the activities of larvae from different stages of development upon the same Light-On stimulus (Table 5); whereas the results of GLMM in this study further explained the details of these differences. First, the LOAs were different between larvae at 3 and 6 dpf due to significant interactions with time (Table 4; ${\beta }_{{I}_{1}}^{(6)}-{\beta }_{{I}_{1}}^{(3)}$: 3.2779; ${\beta }_{{I}_{2}}^{(6)}-{\beta }_{{I}_{2}}^{(3)}$: 36.3283). This indicates the LOAs of these larvae were changing differently over time, as also shown by the left panel of Fig. 3. Second, the LOAs were different between larvae at 3 dpf and 9dpf, both at the main-effect level (Table 4; ${\beta }_{G}^{(9)}-{\beta }_{G}^{(3)}$:0.9693), and the interactions-with-time level (${\beta }_{{I}_{2}}^{(9)}-{\beta }_{{I}_{2}}^{(3)}$: 14.7728). The significant main effect suggests the LOA curves between these larvae should be similar in shape, as demonstrated in the left panel of Fig. 3. The main difference between these curves is that the one for 9-dpf larvae was generally shifted upward (${\beta }_{G}^{(9)}-{\beta }_{G}^{(3)}$: 0.9693). Third, the LOAs were different between larvae at 6 dpf and 9 dpf in both the main effect (Table 4; ${\beta }_{G}^{(9)}-{\beta }_{G}^{(6)}$: 1.1838) and the interactions with time (${\beta }_{{I}_{1}}^{(9)}-{\beta }_{{I}_{1}}^{(6)}$: −2.3585; ${\beta }_{{I}_{2}}^{(9)}-{\beta }_{{I}_{2}}^{(6)}$: −21.5533). This indicates that the LOA curve of the 9-dpf larvae was shifted upward and was changing in a nonparallel pattern compared with that of the 6-dpf larvae, as showed in the left panel of Fig. 3.

**Table 5:** The Hotelling’s T-squared test of Light-On VMR data used in Table 4.

	3 dpf VS. 6 dpf	6 dpf VS. 9 dpf	3 dpf VS. 9 dpf
Test statistic* (p-value)	**4.1605 (<0.0001)**	**6.1705 (<0.0001)**	**5.9348 (<0.0001)**

^*^These results are reproduced from^31^ for comparison.

## Discussion

The locomotor behaviour of zebrafish larvae has been widely used to study neurobehaviour. One reason for this popularity is that these larvae are small and are amenable for high-throughput collection of data from multiple larvae arranged in multi-well plates. The larval activities collected from this arrangement, however, present challenges to statistical analysis. These values are not only measured repeatedly over time, but they are also imbalanced and correlated in time and by location. These statistical issues cannot be dealt with by traditional methods including the t-test and ANOVA. In a previous study, we addressed the time-dependency issue by the Hotelling’s T-squared test^31^. In this investigation, we addressed the data-imbalance problem and location-correlation issue using the GLMM.

The GLMM modeled the relationship between binary responses and explanatory predictors with both fixed and random effects^41^. This approach offered at least two advantages in analyzing VMR data: First, it reduced the degree of imbalance in zebrafish responses by transforming the activity values into binary responses. All non-zero values were transformed to 1, or zero otherwise. For example, all larvae in the right panel of Fig. 1 had mean activities less than 0.075. Many of them actually did not move during any second and had a zero in their response value. This phenomenon resulted in more zero values in responses. When these larval activities were transformed into binary responses, the maximum proportions of moving larvae were close to 0.5, comparable to the proportion of zero values. Second, the GLMM considered the location effect introduced by repeated measurements as a random effect, and explicitly quantified the variations among different locations by estimating the variance of random effect. For example, the variance of random effect in Example 1 was estimated to be 0.1149, indicating that the variations among different wells was 0.1149.

The GLMM had some limitations in analyzing VMR data. First, it only handled binary responses and quantified the probability of larval movement; it could not handle larval displacement. Second, the LOAs from the model might show nonlinear patterns across time (Fig. 2, left and right panel), and the linear and quadratic terms in the model could not capture such patterns. To address the first limitation, we propose that the VMR data should be analyzed by both the GLMM and the Hotelling’s T-squared test. The GLMM quantifies the probability of moving (i.e. how many larvae moved), whereas the Hotelling’s T-squared test defines whether the mean activities (i.e. how much the larvae moved) between two groups are different. Combining these observations would provide a better interpretation of the larval movement. The Hotelling’s T-squared test would also facilitate building a GLMM. When the test finds larval activities significantly affected by certain variables, these variables can be used to build the GLMM. To address the second limitation of GLMM, further analysis should be focused on using smoothing spline ANOVA, a nonparametric model to characterize the nonlinear pattern across time.

To conclude, this study has implemented the GLMM to solve the data-imbalance and location-correlation issues in VMR data analysis. This approach also complements the Hotelling’s T-squared test. Together, they reveal distinctive aspects of locomotor output of a group of larvae induced by light and by different experimental perturbations. This information would facilitate the analysis of activation circuitry that drives locomotor behaviour in zebrafish^42^. Such knowledge may aid translating the interesting findings from neurobiology^1–15^, pharmacology^3, 5–7, 9–12, 16–18^ and toxicology^19–27^ to humans. We recommend the following general data-analysis workflow: (1) Compare the average larval activities of different groups with the Hotelling’s T-squared test; (2) Select significant variables as candidate predictors and apply the GLMM to model the relationship between binary responses and candidate predictors; and (3) Combine the results from (1 & 2) to interpret larval activities. These two statistical approaches therefore have established a statistical framework for VMR analysis that can be generalized to other locomotor behavioural data with similar data structure. This framework is expected to provide new insights into neurobehavioural studies.

## Electronic supplementary material

R-Script
